# Self-assembled 3D Interconnected Magnetic Nanowire Networks for Neuromorphic Computing

**DOI:** 10.1021/acsami.4c22620

**Published:** 2025-03-23

**Authors:** Dhritiman Bhattacharya, Colin Langton, Md Mahadi Rajib, Erin Marlowe, Zhijie Chen, Walid Al Misba, Jayasimha Atulasimha, Xixiang Zhang, Gen Yin, Kai Liu

**Affiliations:** † Physics Department, Georgetown University, Washington, D.C. 20057, United States; ‡ Mechanical and Nuclear Engineering, Virginia Commonwealth University, Richmond, Virginia 23284, United States; § Physical Science and Engineering Division, King Abdullah University of Science & Technology, Thuwal 23955-6900, Saudi Arabia

**Keywords:** 3D nanomagnetism, nanowire networks, 3D information storage, neuromorphic computing

## Abstract

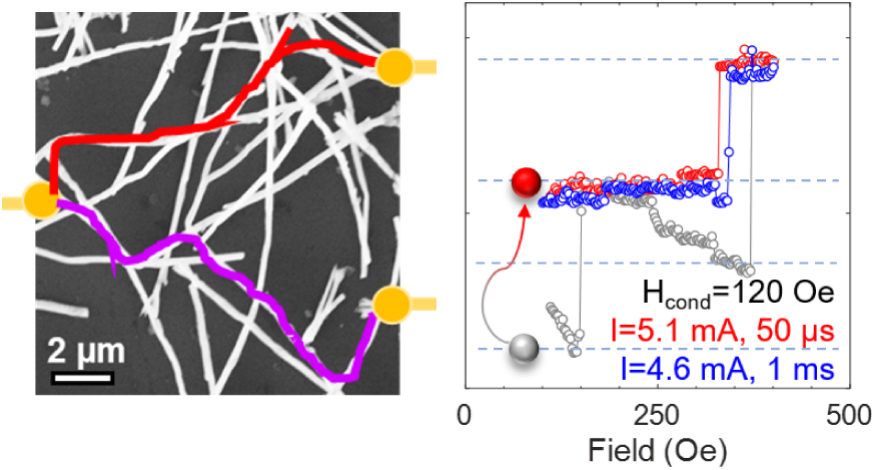

Three-dimensional (3D) nanomagnetic systems offer promise toward implementing neuromorphic computing due to their intricate spin textures, magnetization dynamics, and nontrivial topology. However, the investigation of 3D nanomagnetic systems is often constrained by demanding fabrication and characterization requirements. Here, we present interconnected networks of self-assembled magnetic nanowires (NW) as a novel 3D platform with attractive characteristics for neuromorphic computing. The networks contain multiple unique transport pathways, each hosting discrete magnetization states. These pathways can be selectively addressed, and the magnetic state within them can be electrically controlled by applying current pulses. Consequently, the pathways can serve as synaptic weights, allowing for diverse programming by switching specific sections of the network using current pulses of varying magnitudes and durations. Additionally, unique features such as history-dependent magnetic state switching and interconnected transport paths are observed in these networks. These capabilities are leveraged to illustrate the potential of interconnected magnetic NW networks as reservoir layers in a neural network architecture, highlighting their promise as an efficient platform for neuromorphic computing.

## Introduction

Nanomagnetic devices are promising for multifunctional and energy-efficient neuromorphic hardware^1−3^ as demonstrated in recent examples such as spin-torque nano-oscillator-based spoken digit and vowel recognition schemes,^4−6^ reservoir computing,^7−12^ stochastic computing,^13−15^ and synaptic devices.^16,17^ With the advancement of novel fabrication techniques, recent attention has been drawn to 3-dimensional (3D) magnetic nanostructures, which may provide enhanced functionalities toward implementing nanomagnet-based neuromorphic computing due to their unique structures, intricate spin textures, and nontrivial topology.^18−28^ For example, we previously demonstrated quasi-ordered interconnected nanowire (NW) networks with multiple discrete magnetic states due to the sequential switching of NW sections separated by interconnects, which are promising features to implement multistate synaptic devices.^29,30^

While 3D platforms offer promising characteristics, there are also challenges associated with demanding fabrication and characterization requirements. Here, we explore a novel 3D system of interconnected networks of self-assembled magnetic NWs, offering a straightforward and efficient fabrication process. Previously, self-assembled networks fabricated from nonmagnetic memristive NWs have been studied.^31−33^ Magnetic NW networks add a whole new degree of freedom to control complexity and adaptability, as spin textures, magnetization dynamics, and coupling between NWs can be dialed up not only by modifying the NW geometry but also by leveraging the vast magnetic parameter space to essentially design the energy landscape. Thus, efficient control of such a system holds promise for future low-power neuromorphic applications. In our networks, the interconnected structure enables the formation of multiple unique transport pathways, determined by the presence or absence of NW intersections. Each pathway hosts discrete magnetization states, which can be selectively addressed, and the magnetic states within the pathways can be electrically controlled by applying current pulses of varying magnitudes and pulse widths. Additionally, unique features, such as history-dependent magnetic state switching and interconnected transport paths, are observed in these networks. Thus, the 3D magnetic NW networks closely emulate the self-organized nature of brain architecture, where heterogeneous synaptic connections and complex interactions govern the system functionality. These capabilities are leveraged to illustrate the potential of incorporating interconnected magnetic NW networks in neural network architectures, highlighting their promise as an efficient platform for neuromorphic computing.

## Results

### Sample Fabrication

**Figure 1:**
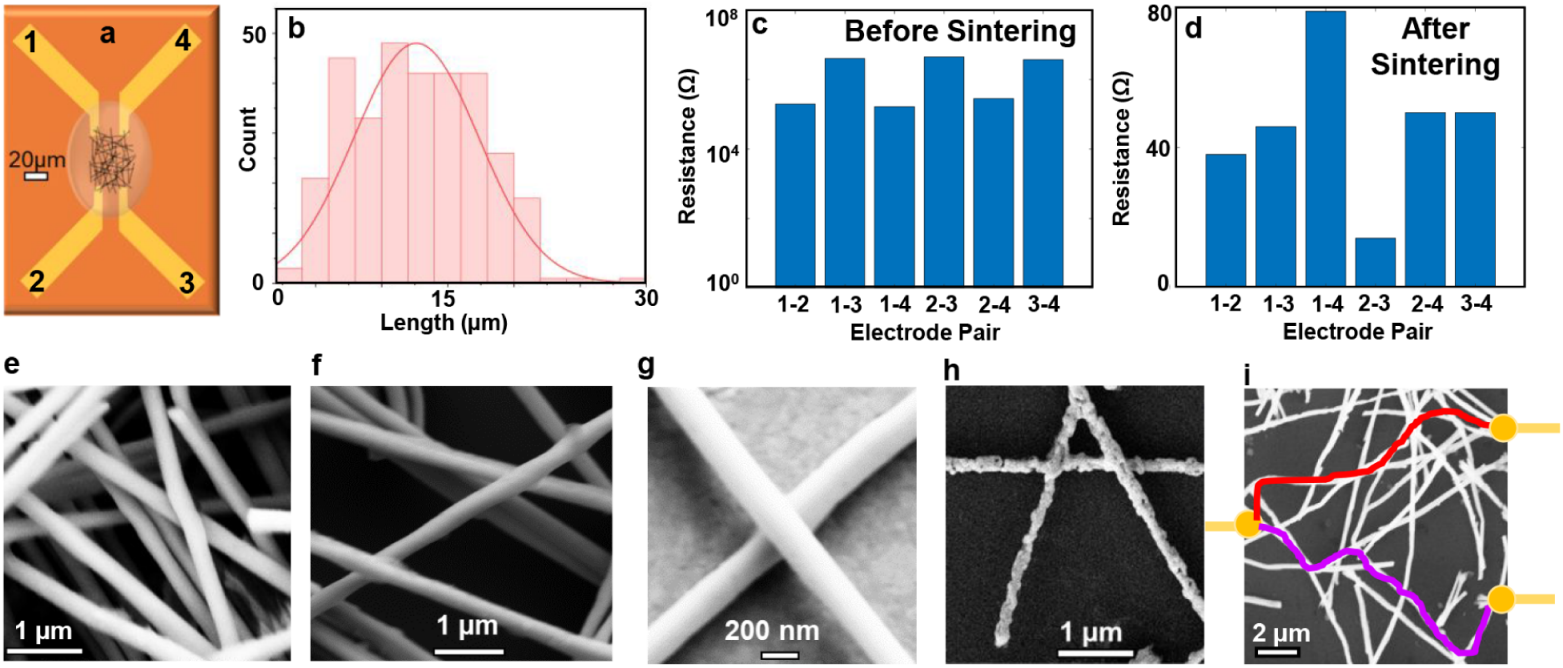
Fabrication and morphology of 3D self-assembled interconnected nanowire networks. (a) Illustration of drop-casting process on prefabricated electrodes. Separations were 20 μm between adjacent electrode pairs 1–4 and 2–3, and 50 μm between diagonal electrode pairs 1–3 and 2–4. (b) Length distribution of the NWs in a network, (c,d) Electrical resistance measured between different electrode pairs before and after sintering, respectively, and (e–h) SEM images of different Ni NW networks. Panels (e) and (f) show the random arrangement of layers of NWs. Panels (g) and (h) show a zoomed-in view of isolated intersections with a physical bond after sintering at 350 °C and 420 °C, respectively. (i) Schematic of multiple pathways within an interconnected NW network.

To fabricate the self-assembled networks, Ni NWs were first synthesized by electrodeposition into anodized aluminum oxide (AAO) membranes.^34,35^ Details of the synthesis are provided in the Materials and Methods section. The membranes were then dissolved, and the harvested NWs were drop-cast on prefabricated electrodes (Figure 1a). The NW diameter follows the pore distribution of the AAO template of 200 nm with a 10% variability. The mean length after drop-casting was found to be 12.5 μm (Figure 1b). After drop-casting, the NW networks were not electrically conducting and exhibited high resistance. Next, sintering was performed at a temperature ranging from 320 to 420 °C with several oxidation and reduction cycles,^34,35^ after which the resistance decreased significantly, from MΩ to tens of Ω (Figure 1c,d). This indicated that sufficient NWs had ohmic contacts with one another and formed a conducting network. Scanning electron microscopy (SEM) image of the network revealed that the NWs were randomly arranged in a mostly in-plane fashion, where many layers of NWs lay on top of one another, and each NW was in contact with one or more wires of the complex network (Figure 1e–h). Zoomed-in view of isolated intersections showed that NWs were overlapping and formed a physical bond (Figures 1g,h). The resistance of the sintered network measured using four electrodes marked by numbers 1 through 4 showed varying values (Figure 1c,d). This means that while there are numerous intersections in the whole network, only a subset of intersections within the path of least resistance is probed between each electrode pair. Hence, a unique transport pathway formed between each electrode pair determined by the presence or absence of NW intersections,^36^ similar to the illustration in Figure 1i. As a result, the propagation of magnetic states within the different pathways of the networks can mimic the way the signal travels through biological neural networks. The resistance values depend on the network properties such as the length of the network, the density and size of the NWs, and the number and degree of intersections. These properties can be controlled by optimizing the fabrication conditions. For example, the sintering temperature can be varied to control bonding at the intersections. The networks shown in Figure 1g,h were sintered at 350 and 420 °C, respectively. The degree of intersection between the NWs in the network increased after sintering at higher temperatures. However, the NWs sintered at the lower temperature remained smooth while maintaining conducting paths through the networks. These networks were found to be more suitable to establish multiple pathways and induce current-controlled switching. Thus, a sintering temperature of 350 °C was used for the rest of the study. The NWs in the network were stable, and the resistance measured between electrode pairs remained stable for at least 3 weeks after fabrication.

### Magnetoresistance and Magnetization Reversal

**Figure 2:**
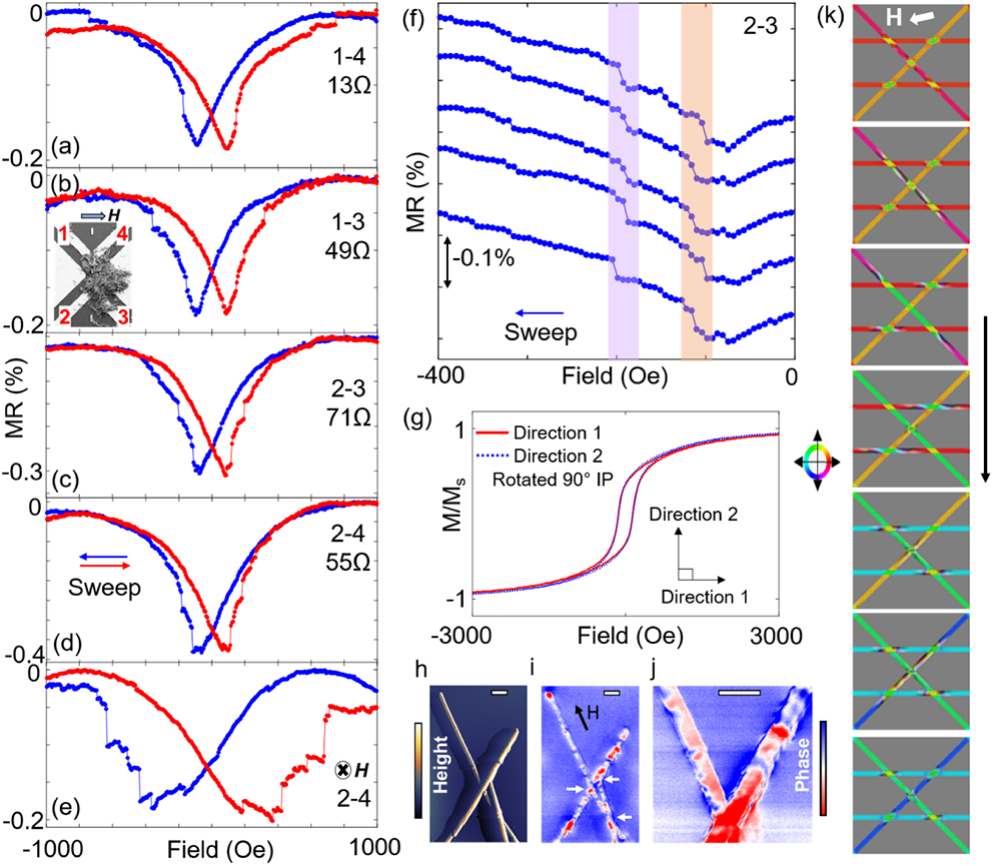
MR measurement and magnetization reversal in the network. (a–d) MR curves measured using two adjacent electrode pairs (1–4 and 2–3) and two diagonal electrode pairs (1–3 and 2–4) with a magnetic field applied as shown in panel (b). All the MR curves are unique and show multiple discrete jumps indicative of step-by-step switching. (e) MR curve measured using the same electrode pair in (d) when the applied field is out of the plane. (f) 5 individual MR measurements using the same electrode pair showing consistent behavior. The shaded areas highlight two particular jumps occurring at nominally the same field. (g) Hysteresis loops of a network with applied field along two orthogonal in-plane directions. (h,j) MFM imaging of different networks at remnant state after saturation in a 1T in-plane magnetic field, with the lateral scale bars showing 2 μm. Panel (h) shows the topography, and panels (i) and (j) show the phase information under different magnifications. The pinned domain walls are marked by white arrows in panel (i). Panel (j) is a zoomed-in view of part of panel (i). Height and phase scale bars are 1.5 μm and 1.6°, respectively. (k) Micromagnetic simulation of magnetization reversal under an increasing reversal field showing step-by-step switching.

To gain insights into the microscopic magnetization reversal mechanism in the networks, magnetoresistance (MR) measurements were performed which is effective in probing complex geometries.^37−39^ The MR behavior in the network originates from the anisotropic magnetoresistance (AMR) which depends on the relative alignment between the magnetization and the current flow: *R* = *R*_T_ + Δ*R*cos^2^ α. Here, α is the angle between the magnetization and the current, and *R*_T_ is the resistance when the magnetization and the current are perpendicular to each other. This means that in the NWs, where the current flows along the wire axis, magnetization parallel (perpendicular) to the NW axis would yield a high (low) resistance. MR was measured using different electrode pairs connecting the network sweeping the magnetic field in the range of ±1.2T. All MR curves exhibited many discrete jumps during field cycling, suggesting step-by-step magnetization switching (Figure 2a–d). Furthermore, when MR curves were measured using different pairs of electrodes, unique features emerged. This was manifested in the variation of the number and location of MR jumps for each electrode pair (Figure 2a–d). Thus, in each transport path, different sections of the network switched at different magnetic fields due to variations in the local energy landscape. More cases are illustrated in Figure S2. Additionally, when the direction of the applied field was changed, despite using the same electrode pairs as in Figure 2e, the switching behavior differed significantly in the number and locations of MR jumps. Consistency of the MR response was ensured by measuring multiple MR curves by using the same electrode pairs (Figure 2f).

Magnetometry performed in two orthogonal in-plane directions exhibited almost identical hysteresis loops, with similar remanence and coercivity (Figure 2g), due to the random, isotropic nature of the magnetic NW layouts. The effect of individual intersections was not evident in these magnetometry measurements. This indicated that the discrete jumps seen in an MR measurement arise from a few specific NWs within the transport path probed by the electrode pair and correspond to switching in only those NWs. Magnetization switching in cylindrical NWs is usually mediated by domain wall (DW) propagation. Thus, the MR jumps could result from two possible scenarios: either each NW switches independently through DW propagation along its length or specific DW pinning sites within the network cause certain sections, defined by these pinning locations, to switch in discrete steps. Independent switching of each NW appears unlikely, as the number of MR jumps varies with changes in the field direction (Figure 2f). If each MR jump corresponded to a single NW, then this number should have remained the same. Moreover, we previously observed DW pinning at intersections in interconnected NWs using off-axis electron holography.^30^ Similarly, DW pinning at the constriction of geometrically modulated cylindrical Ni NWs has been shown.^40^ In the networks studied here, magnetic force microscopy (MFM) was used to image the magnetic state (Figure 2h). Sharp contrasts were found at NW intersections (Figure 2i,j), indicating pinning of DWs. This suggests the presence of an energy minimum for the DWs at the intersection, similar to the DW pinning found previously in interconnected Co NWs.^30^ Additionally, alternating bright and dark contrasts were seen along the NWs (Figure 2i,j), possibly due to longitudinally magnetized domains pointing in opposite directions.^41^ More MFM images are shown in Figure S3. Therefore, DW pinning at the NW intersections and propagation are the likely magnetization reversal mechanisms. Considering pinning sites at the NW intersections, micromagnetic simulations were performed. The network size was restricted to a few NWs limited by the computational resources required.^42,43^ The simulations showed that starting from a state with magnetization pointing along the NW axis, with increasing magnetic field the domains start to merge, propagate, and eventually get pinned at different intersections during magnetization reversal (Figure 2k). Thus, the magnetization reversal proceeded in a step-by-step fashion which manifests in the MR jumps. Collectively, the propagation of discrete magnetic states throughout the network governed by its topology offers a potential way to encode information within the network, not solely in the form of discrete magnetic states, but also through the routes they traverse.

### Control of Magnetic States

**Figure 3:**
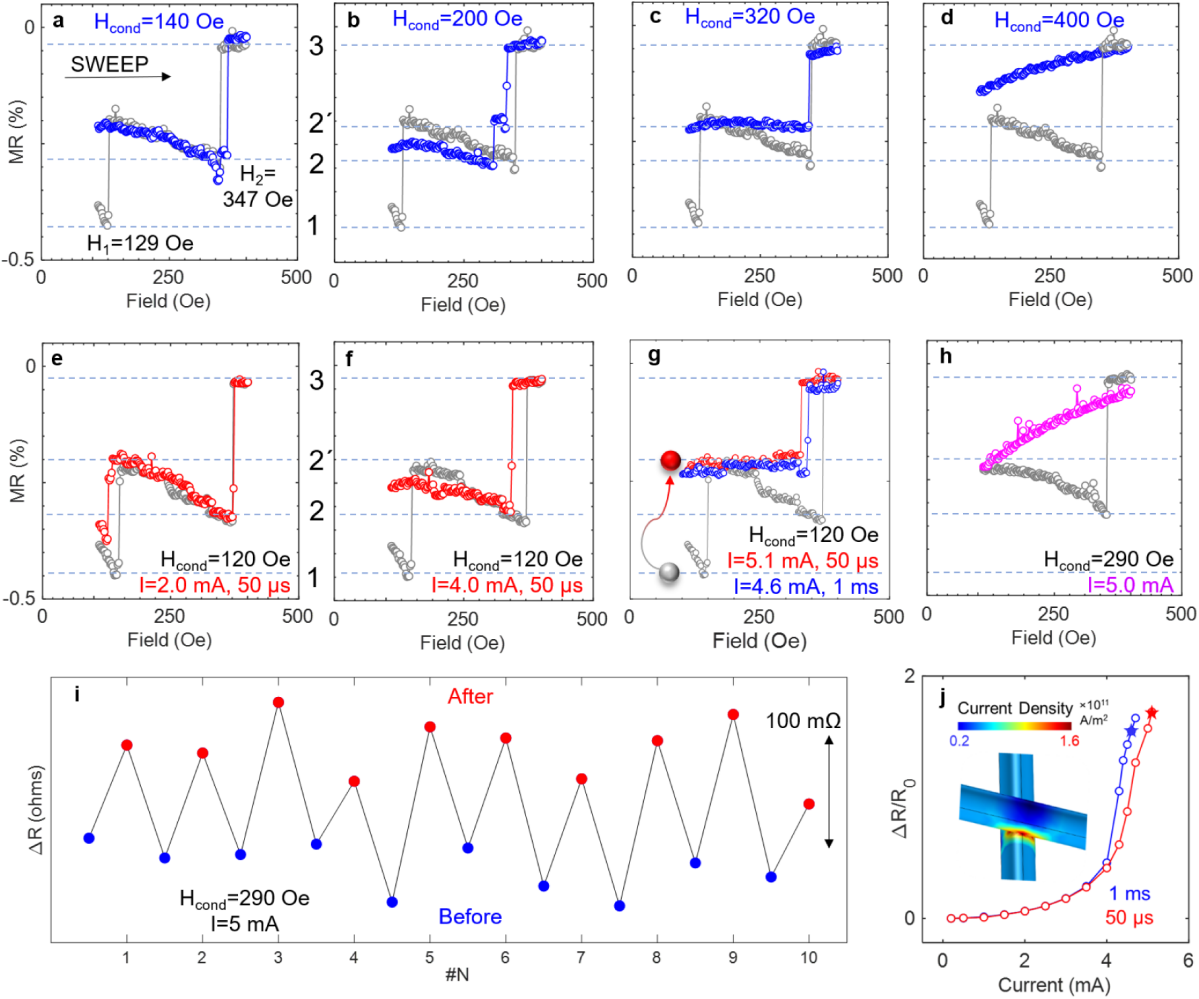
Control of magnetic states. (a–d) MR curves for different conditioning fields showing stabilization of nonvolatile magnetic states. The gray curve shows the section of the ascending major loop with discrete states marked as 1–3. Minor MR curves for current pulses (e–g) with different magnitudes and pulse widths at *H*_cond_ = 120 Oe, (h) with *I* = 5.0 mA, 50 μs at *H*_cond_ = 290 Oe. (i) Resistance before and after application of current pulse showing repeatable switching and resetting between two states. (j) Resistance increases due to Joule heating during current pulse application. COMSOL simulation in the inset shows a high current density concentrated at the intersection between NWs.

The aforementioned step-by-step switching behavior could be controlled and modulated by using external stimuli. First, we achieved this in another device with different sequences of the applied field while measuring the resistance between electrodes 1–2 (*R* = 44 Ω). In Figure 3a, the gray curve shows the ascending major loop in the field scanning range from 95 Oe to 400 Oe measured at 2 Oe intervals. Two clear jumps were observed at *H*_1_ = 129 and *H*_2_ = 347 Oe, suggesting the existence of at least three well-distinguished states, marked 1–3. To probe these states, (i) the network was first negatively saturated at −1T, (ii) then reduced to a positive conditioning field (*H*_cond_), and (iii) brought back to the remnant state at zero field. Finally, (iv) MR was measured by scanning the field from zero to positive saturation. If the magnetic states were discrete and nonvolatile, the network would have stabilized at different remnant states when *H*_cond_ > *H*_*i*_ (the switching field for the *i*th jump), causing jumps 1 – *i* to disappear during the subsequent field sweep in step iv. This was indeed the case as shown in Figure 3a–d. For instance, at *H*_cond_ = 140 Oe (larger than *H*_1_ = 129 Oe), jump 1 disappeared during the final scan (blue curve in Figure 3a). At *H*_cond_ = 400 Oe, which exceeds *H*_2_ = 347 Oe, no jumps were observed (Figure 3d). Interestingly, after the first switch, the magnetization reversal path deviated from the major loop. This is shown in Figure 3b, where an extra jump was observed for *H*_cond_= 200 Oe. In other words, switching from state-2 to state-3 now proceeded through an additional discrete state, marked as 2'. This state could also be accessed with the appropriate field conditioning (Figure 3c). These results illustrate the intricate energy balance and strongly history-dependent reversal behaviors, attractive to design complex neuromorphic systems.

To implement practical multistate memristors and synaptic devices, nonvolatile control of magnetic states via electric currents is desired, which we show next. The network was first initialized in a state where a certain section was on the verge of switching (*H*_cond_= 120 Oe, close to *H*_1_ = 129 Oe) and then current pulses of different magnitudes were applied. Finally, the magnetic field was brought back to zero, and the MR was measured to positive saturation. When current densities were below a threshold (*I* = 2 mA), the same MR jumps as the reference curve (*H*_cond_= 120 Oe, *I* = 0 mA) were observed (Figure 3e). However, larger currents caused deviations from the reference curve, indicating nonvolatile magnetization switching. For example, at 4 mA, jump 1 disappeared, showing a switch from state-1 to state-2 (Figure 3f). Similarly, for *I* = 5.1 mA, state 2' was reached from state-1 (Figure 3g). For *H*_cond_ = 290 Oe and *I* = 5.0 mA, switching from state-2 to state-3 was achieved as evidenced by the disappearance of all the discrete jumps (Figure 3h). For a pulse width of 1 ms, *I* = 4.6 mA was needed to drive the magnetization from state-1 to state-2', which is lower than the current required for a pulse width of 50 μs (Figure 3g). Thus, the network could be driven to different metastable states by controlling the magnitude and duration of the current pulses. Comparing the field- and current-induced MR curves (Figure 3c and g), the maximum effective magnetic field induced by the current application can be estimated to be ∼200 Oe. This switching was nonvolatile and the magnetic state persisted after the withdrawal of current pulses and the conditioning field. This information can be erased by saturating the network at a high magnetic field, after which the initial reference MR curve can be reproduced (Figure S4). The reproducibility of the switching was ensured by directly measuring the resistance of the network multiple times before and after the application of a 5.0 mA current pulse at *H*_cond_ = 290 Oe (Figure 3i). Furthermore, such nonvolatile switching was observed in various networks (Figure S6). The switching pathways in each network are determined by its distinct morphology and connectivity, allowing the number of states and the transitions between them to be configured based on these aspects.

Previously, it was shown that different factors can contribute to the switching process such as thermal depinning, spin transfer torque (STT), Oersted field, etc.^44−46^ In our experiments, the resistance of the network increased (Δ*R*) from the initial value (*R*_0_) during the current pulse application, indicating Joule heating (Figure 3j). Note that the resistance returned to the initial value after the withdrawal of the pulse. For a similar level of heating (i.e., Δ*R*/*R*_0_, marked by stars in Figure 3j), switching to the same state was observed (Figure 3g) which also supports Joule heating-induced switching. The temperature increase is estimated to be ∼260 °C for these cases. The intersection between NWs can experience even more intense heating where a large current density was found to be concentrated from COMSOL simulation (Figure 3j, inset). Such localized hotspot creation at NW intersections is consistent with thermal microscopy studies in other interconnected metallic NW networks.^36^ Previously, local Joule heating induced reduction of saturation magnetization, reversed DW nucleation, and subsequent magnetization switching was shown in single cylindrical Ni NW.^47^ In diameter modulated Ni NWs, current-induced Joule heating resulted in reversed magnetic domains with DW pinned at the notches.^40^ Torrejon et al. showed that thermal-gradient-driven spin current generation could drive DWs to a hotter region of the sample.^48^ Thus, in our networks, DWs could move to different intersections as a consequence of current-pulse-induced localized heating, resulting in the switching of network subsections.

## Discussion

**Figure 4:**
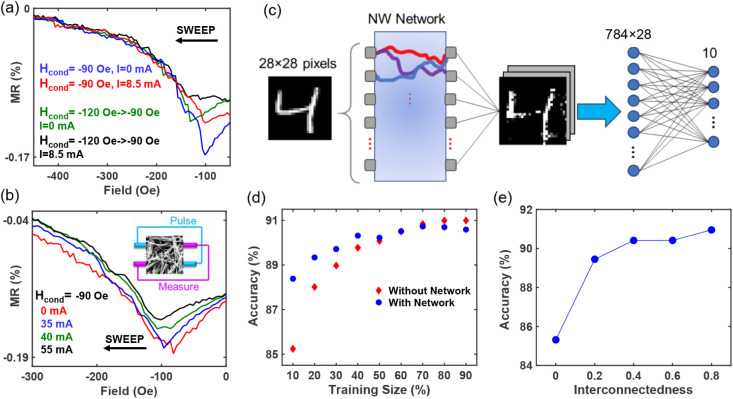
Handwritten digit classification task. (a) Switching to different magnetic states when the same conditioning field and current were used, but with different initial field sequences. (b) Coupling of two transport pathways showing switching can be achieved in one electrode pair while current pulses are applied in another pair. (c) Neural network architecture where the NW network is used as a reservoir-like layer. (d) Evolution of accuracy with training data size showing improvement with the NW network. Interestingly, the best improvement was achieved for the smallest data size. (e) Variation in accuracy as the level of interconnectedness among pathways was adjusted.

These measurements demonstrate that nonvolatile switching of multiple magnetic states can be effectively achieved using electric currents, which is promising for storing synaptic weights. Furthermore, different field and current sequencing could be utilized to manipulate switching to different magnetic states in the networks. For instance, in Figure 4a, using the same conditioning field (−90 Oe) and current (8.5 mA), switching to two distinct nonvolatile states was achieved by varying the initial conditioning. This memory could allow the network to handle sequential data required in applications such as time series prediction and reservoir computing. Moreover, unlike crossbar arrays, resistance changes in the networks should not be dictated by single memristive elements at a given junction but rather by the topology of the network and interconnectivity of NWs. Due to this interconnectedness, switching in a network subsection between one set of electrodes can be achieved by applying a current pulse along a different set of electrodes (Figure 4b). This coupling can be attributed to the common transport path shared between two pairs of electrodes and can be optimized by controlling the extent of the shared path (Figure S8).

The network’s properties were utilized to demonstrate a classification task on the MNIST data set—a collection of 28 × 28 grayscale images of handwritten digits (0–9)—where the network functions as an untrained reservoir layer, nonlinearly transforming the features of the original inputs into higher-dimensional parameter space such that they are easier to separate using networks with lower complexity.^4,49^ The architecture is shown in Figure 4c. The NW network is modeled with 28 input and 28 output nodes, fully connected to form 28 × 28 unique pathways. The MNIST images were converted to sequential data, with each input corresponding to a specific path between input and output nodes. For each input, output from all 28 nodes was taken to increase the dimensionality. These processed images were then fed into a simple fully connected layer with 784 × 28 inputs and 10 outputs where the standard backward propagation process was used for training.

The NW network paths were modeled using 10 discrete resistance states that were nonlinearly distributed along a sigmoid function. This is in line with the experimental observation of change in MR vs current magnitude (Figure S9). Additionally, variations in slope, initial resistance, and horizontal shift across different pathways were included to mimic the uniqueness of each transport path between each input and output node. The resistance of each path was defined by
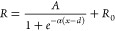
1

Here, *A* is the amplitude and α is the slope of the sigmoid function, *d* is the horizontal shift, and *R*_0_ is the initial resistance of the pathway. The mean value of *R*_0_ was set to 20 Ω, with a 1% spread in initial resistance among the paths (Figure S9). *A* was set to be either 0.2 or 2, resulting in an MR ratio of approximately 1% or 10%, respectively. The 1% MR change is comparable to the value in the present Ni NWs, while higher MR ratios, such as 10%, can be achieved using the giant magnetoresistance effect in multilayered nanowires.^50,51^

To account for the network’s interconnectedness, as shown in Figure 4b, neighboring paths were assumed to share common transport paths with closer electrode pairs sharing more strongly.
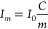
2

Here, *I*_0_ is the primary input to a pair of electrodes, *I*_*m*_ is the effective input to other pairs due to the primary input, *C* is a constant that reflects the interconnectedness of the paths, and *m* is a dimensionless index between 1 and 28 that represents the distance between output nodes sharing the same input node or vice versa. Thus, if an input current pulse *I*_0_ is applied to one path, *CI*_0_ will flow through the immediate neighboring path and decrease by 1/m for the paths farther away from each other. Lastly, the network’s memory was designed to reflect the history-dependent switching behavior, as shown in Figure 4a. For simplicity, we assumed that reaching the next state requires one-third less current than reaching the first state.

Notable improvement was observed when the network was employed with a 10% MR ratio (Figure 4d), while that for 1% MR showed no appreciable improvement, in part due to signal-noise limitations. This suggests that improving the MR ratio and achieving greater uniformity in resistance pathways will be key to practical applications. In the simulations, the best improvement was achieved when the training data set size was the smallest. Achieving high accuracy with minimal data is particularly beneficial for edge computing applications. Figure 4e depicts the variation in accuracy as the level of interconnectedness (*C*) between pathways was adjusted, highlighting the role the network properties play in performance improvement and suggesting further gains could be achieved with enhanced interconnectedness.

## Conclusions

In conclusion, we demonstrated a novel 3D nanomagnetic platform, interconnected magnetic NW networks, with promising attributes for the implementation of neuromorphic functionalities. Multiple pathways can be selectively addressed in the network, each with uniquely distributed and electrically controllable discrete magnetic states. These characteristics were utilized to test the MNIST problem, demonstrating a performance improvement with the NW network. This network behavior, with distinct pathways and unique magnetoresistance responses across different paths, shares similarities with that of the human brain. In the brain, individual unique synaptic connections contribute to larger neural circuits that operate collectively to perform complex cognitive functions. Similarly, in this nanowire network, each pathway, consisting of multiple intersections, exhibits unique behavior, with interactions among them. Future research could explore precise reversal mechanisms at individual NW intersections, how these translate to large-scale network behavior, and additional approaches for achieving large-scale integration. Overall, this study provides a foundation to inspire further work in designing 3D multifunctional neuromorphic systems using this platform.

## Materials and Methods

### Synthesis

The nanowires were formed by the electrodeposition of nickel into porous anodized aluminum oxide (AAO) templates.^34,35^ Each AAO template was sputter-coated with a thin layer of metal, typically copper, as the electrode in the deposition process. The electrolyte was composed of 1.5 M nickel sulfate and 0.6 M boric acid in deionized water with a pH of 3.5. The nanowires were deposited with an applied potential of 1 V relative to a Ag^+^/AgCl reference electrode, using a Princeton Applied Research Potentiostat 263A. The nanowires were liberated from the AAOs through chemical etching and several cycles of washing. The networks were examined by a ZEISS-SUPRA A55 Scanning Electron Microscope.

### Magnetometry

Magnetic properties of the networks were measured by vibrating sample magnetometry (VSM) at room temperature with an external field of up to 1.2 T after the sintering process.

### MR Measurement

Magnetoresistance (MR) measurements were performed by applying current pulses using a Keithley 6221 source and reading the voltage with a Keithley 2182 nanovoltmeter. Different current magnitudes were used for measurement depending on the resistance of the network with a 12 ms pulse width. For current-induced switching experiments, larger magnitude pulses with varying pulse widths were applied. The period of the current pulse was 83 ms in all cases.

### Magnetic Force Microscopy

MFM images were obtained at room temperature and atmospheric pressure with a Bruker Dimension Icon AFM system with Bruker MESP-HM probes. To confirm that there were no tip-induced effects, some areas were scanned twice (up and down). The two scans produced similar images. The nominal cantilever frequency, lift height, and scan rate were 75 kHz, 80 nm, and 0.2 Hz, respectively. The dark and bright contrasts along the NWs correspond to the attraction and repulsion of the magnetic tip magnetized perpendicular to the substrate.

### Micromagnetic Simulations

We used Mumax3 to simulate a simplified four-wire network, as shown in Figure 2k, including two parallel and two diagonal wires. The simulation space was 4000 × 4000 × 250 nm^3^, and the cell size was 5.2 × 5.2 × 7.8 nm^3^. Each nanowire was 200 nm in diameter with an infinite length. The two parallel wires were 1.5 μm apart. We assumed exchange stiffness (*A*_ex_) and saturation magnetization (*M*_s_) to be 9 pJ/m and 4.9 × 10^5^ A/m, respectively.^52^ The five intersection areas were considered to have uniaxial anisotropy (1.0 × 10^6^ J/m^3^) to mimic a realistic pinning scenario. As an extreme case, we purposely set the anisotropy axis to be perpendicular to the long axes of the intersections, with the anisotropy axis of the center intersection being along the vertical direction. The stable magnetization state of the wires was simulated by energy minimization at each field step as the magnetic field sweeps from positive to negative saturation.

## Supporting Information Available

X-ray diffraction of sintered network, examples of more MFM images, examples of MR measurement of different networks, re-saturation, repeatability of current-induced switching, examples of current-controlled switching, estimation of temperature increase, COMSOL simulation, neural network simulation (PDF)
