# Supplementary material for: Self-assembled 3D Interconnected Magnetic Nanowire Networks for Neuromorphic Computing
Source: ACS Appl Mater Interfaces. 2025 Mar 23;17(13):20087–95. doi: 10.1021/acsami.4c22620 (PMC11969432; doi:10.1021/acsami.4c22620)
Supplement: Supplementary file 1 — am4c22620_si_001.pdf [file am4c22620_si_001.pdf]

Supporting Information

Self-Assembled 3D Interconnected Magnetic Nanowire Networks for Neuromorphic Computing

Dhritiman Bhattacharya[1*], Colin Langton[1], Md Mahadi Rajib[2], Erin Marlowe[1], Zhijie Chen[1], Walid Al Misba[2], Jayasimha Atulasimha[2], Xixiang Zhang,[3] Gen Yin[1*], and Kai Liu [1*]

[1]Physics Department, Georgetown University, Washington, DC 20057, USA

[2]Mechanical and Nuclear Engineering, Virginia Commonwealth University, Richmond, VA 23284, USA

[3]Physical Science and Engineering Division, King Abdullah University of Science & Technology, Thuwal 23955-6900, Saudi Arabia

*Corresponding authors. E-mail: dhritiman.bhattacharya@georgetown.edu, gen.yin@georgetown.edu, and kai.liu@georgetown.edu

X-Ray Diffraction

During the sintering process, the network undergoes several oxidation and reduction cycles. We performed X-ray diffraction (XRD) on a sample sintered at 320°C for two cycles, which shows primarily metallic Ni peaks. This indicates that the sintering process did not appreciably oxidize the NWs.

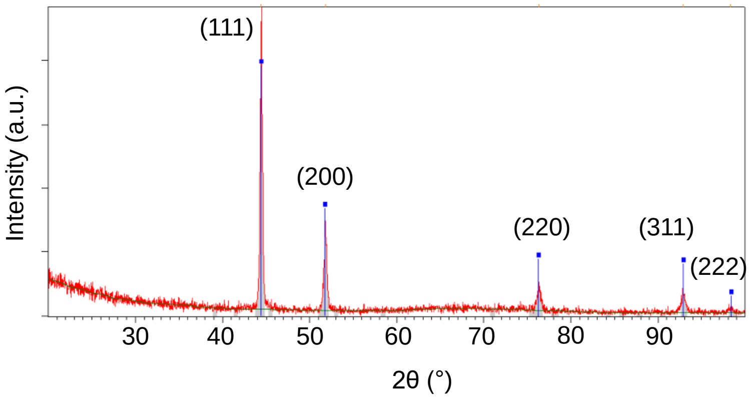

Figure S1. X-Ray Diffraction*. Gonio scans of NW networks after sintering showing only Ni peaks.*

EXAMPLES OF MORE NETWORKS

Figure S2 illustrates magnetoresistance (MR) measurements from 3 different devices which further corroborate step-by-step switching in these networks as well as unique MR behavior determined

by topology and morphology of each network. Network resistance and electrode pairs used for MR measurements are shown in each panel.

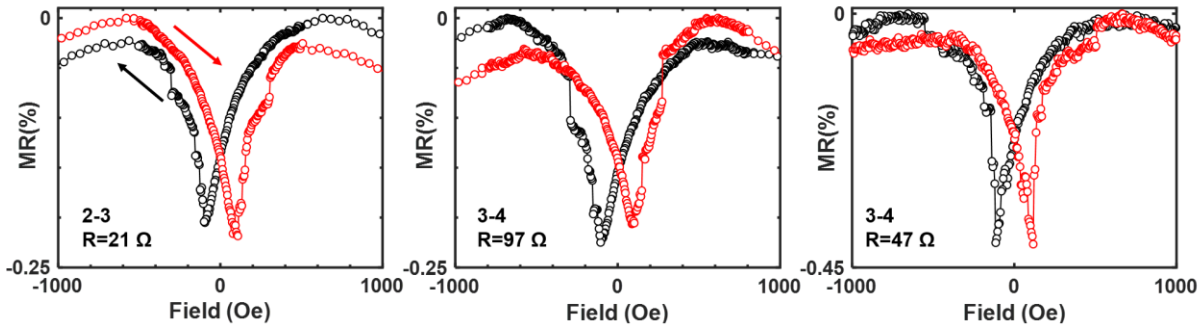

Figure S2. Magnetoresistance (MR) measurement in 3 different Ni NW networks. *These measurements show step-by-step switching and unique MR behavior for each network.*

EXAMPLES OF MORE MFM IMAGES

Figure S3 shows example of more MFM images. Here panels a and c are topography images, while b and d are phase images. These images were taken after saturating the networks in a 1T in-plane magnetic field. Multiple domains and pinned DWs at the intersection were found in both cases as shown in Figure 1 in the main text.

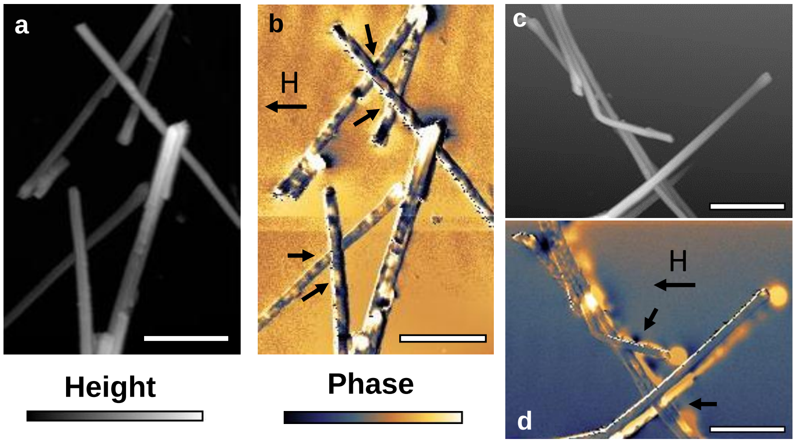

Figure S3. MFM imaging of the magnetic states in different networks. *These images show presence of multiple magnetic domains and pinned domain walls at the intersection between NWs in each network. Lateral scale bars are 5 µm in all panels. Height scale bars are 1 and 1.5 µm in panels (a) and (c), respectively. Phase scale bars are 0.3° and 1° in panels (b) and (d), respectively.*

RE-SATURATION

To show that there was no irreversible change in the network during current pulse application and that current pulse induces magnetic state switching, MR response was first measured before applying a current pulse and compared with the case when the network was re-saturated at a high field after applying a current pulse. Figure S4 shows two MR curves which were measured for the same conditioning field (-105 Oe) with no current. In between these two measurements, a current-induced switching experiment was performed (conditioning field of -105 Oe, and a 7 mA, 50 μs current pulse) and subsequently the network was saturated in a 1 T field. The curves are essentially the same, with all the discrete MR jumps present in both cases. Note that, there is a slight change in the switching field for the MR jump near -500 Oe, which is due to the stochastic nature of the switching in the network. These measurements confirm that there was no permanent change due to current pulsing. Such MR measurements before and after re-saturation were performed periodically during the experiments to ensure the integrity of the networks throughout the experiment.

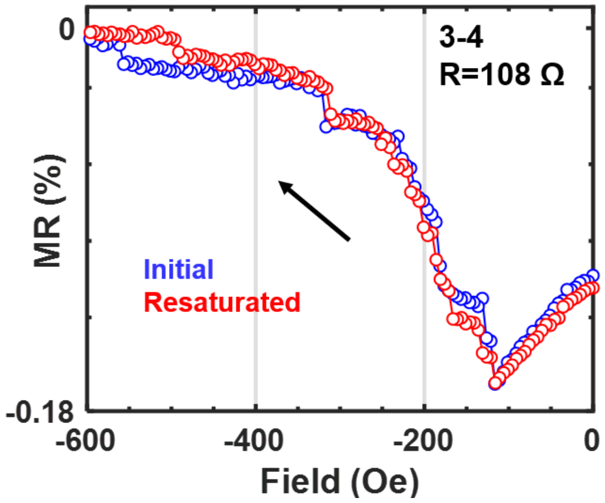

Figure S4. Magnetoresistance (MR) measurement of a Ni NW network from a saturated state before and after applying current pulses*. The MR curves are the same which proves there was no irreversible change in the network and that the current pulse induced magnetic state switching.*

REPEATABILITY OF CURRENT INDUCED SWITCHING

Figure S4 shows an example of repeatability of current induced switching where two different instances exhibited the same MR behavior (i.e. switching to the same magnetic state) after

applying a current pulse of 7 mA magnitude and 50 μs pulse width. Note that, this is a representative case and many such repeatability tests were carried out.

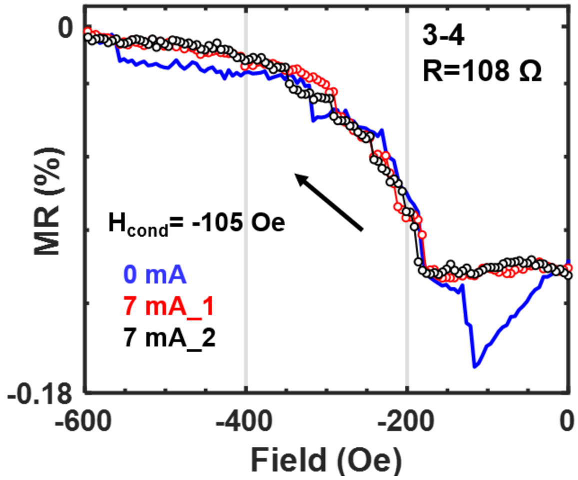

Figure S5. Repeatability of current induced switching in a Ni NW network*. The MR curves after applying the same current pulse shows the same behavior which proves the repeatability of current induced switching.*

EXAMPLES OF CURRENT CONTROLLED SWITCHING

Figure 4 in the main text shows current controlled switching in a network. Here, Figure S6 shows this switching in 3 other optimized networks by measuring MR before and after applying current pulses. The magnitude of the current needed to induce switching was found to vary, which may be related to the topology of the network.

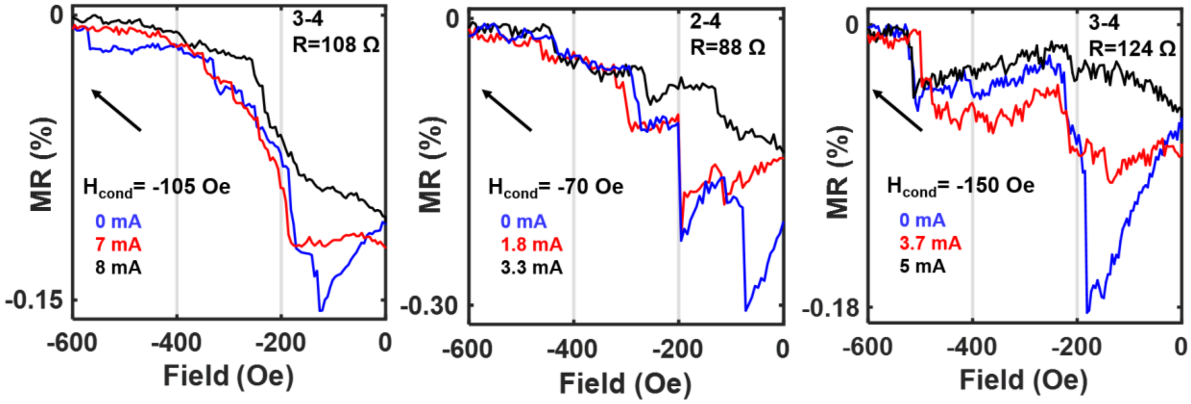

Figure S6. Current induced switching in 3 different Ni NW networks*. The MR curves measured for different current magnitudes show that current-induced switching can be achieved consistently in different networks.*

ESTIMATION OF TEMPARATURE INCREASE

Temperature dependence of a resistor can be approximated. by $\Delta R/ R_0=\alpha\ \Delta T$, where α is the temperature coefficient of resistance and ΔT is the temperature increase. Considering $\alpha = 6 \times 10^{-3}$ $°C^{-1}$ for Ni[1], the temperature increase is estimated to be ~260°C for the cases shown in Fig. 4b.

COMSOL SIMULATION

COMSOL simulation was performed considering a geometry with two NWs (200 nm diameter, 20 µm length) intersecting each other. The NWs had 20 nm overlap at the intersection. One end of each NW was taken to be ground and the other was considered a terminal with a 1 mA input current (Figure S7).

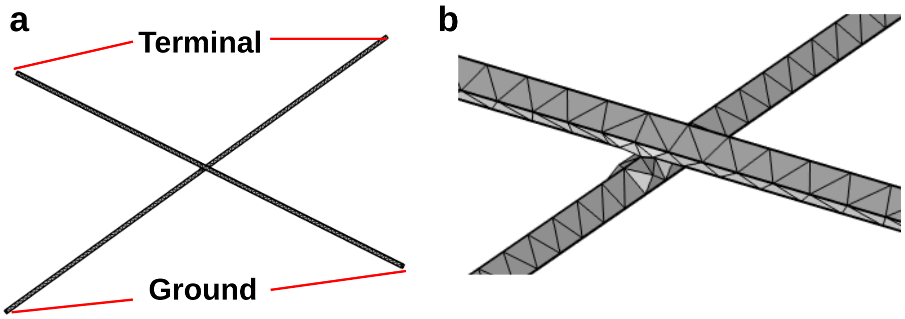

Figure S7. COMSOL Simulation*. (a) Simulated geometry and electrical connections for current density calculation. (b) Zoomed-in view of the intersection with mesh used in the simulation.*

INTERCONNECTEDNESS

In the main paper, coupling between two paths is shown where large current is needed in one path to influence the other path. This indicated a low interconnectedness. In Figure S8, MR behavior of two highly interconnected paths are shown. Here, same current pulse of 5mA amplitude applied using electrode pair 1-3 caused comparable levels of changes in MR measured using electrode pairs 1-3 and 3-4. These measurements show that the different paths in the network can have different levels of interconnectedness determined by the topology of the network. Therefore, this could be controlled by tuning the density of NWs and number of intersections in the networks.

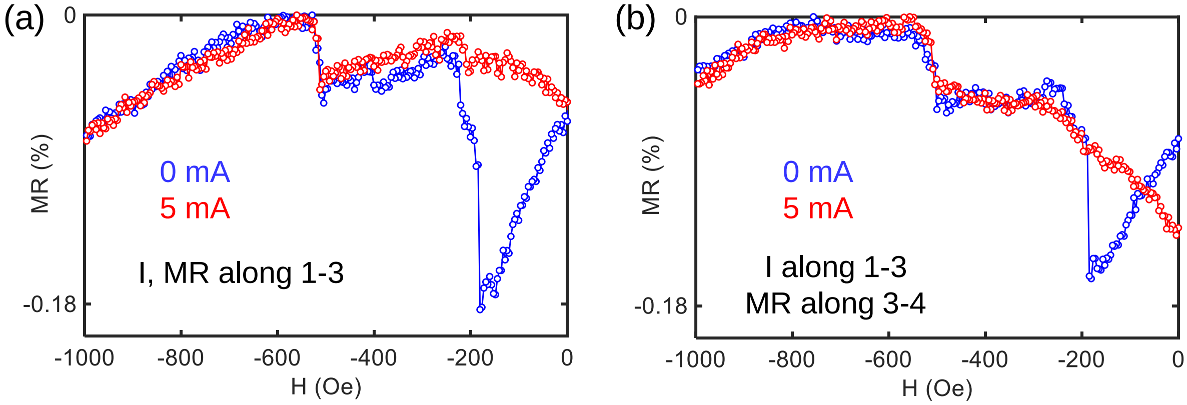

Figure S8. Interconnectedness in the network. *MR behavior measured using electrode pair (a) 1-3 and (b) 3-4, before and after applying 5 mA current pulse. In both cases, current pulse was applied using electrode pair 1-3 which caused discrete switching in both paths indicating high interconnectedness.*

NEURAL NETWORK SIMULATION

Measurements from 40 different networks show that the overall resistance follows a normal distribution with a mean of 40 Ω and a standard deviation of 20 Ω (Fig. S9a). This distribution was obtained without any specific efforts to achieve uniform resistance. A tighter distribution could be realized by optimizing the fabrication process. In the simulations, the mean resistance was set to 20 Ω, with an initial resistance spread of 1% among the paths. The discrete resistance

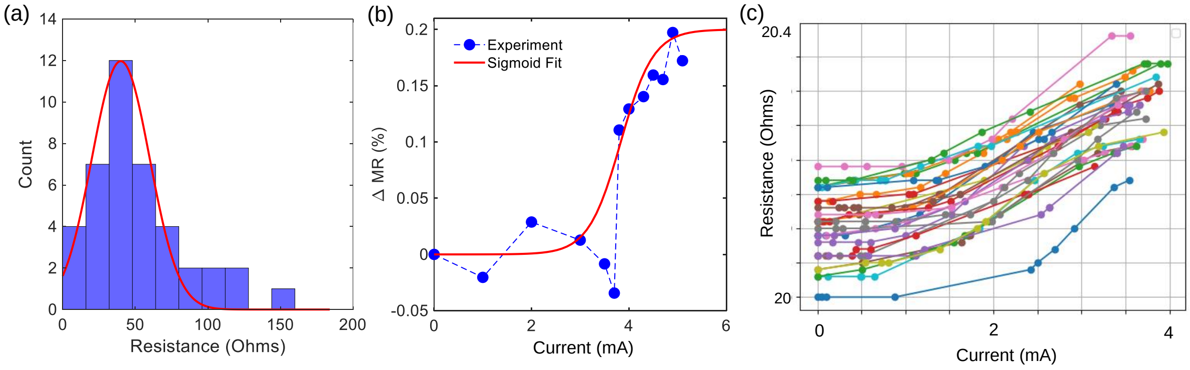

Figure S9. Modeling of the NW network paths *(a) Histogram of resistance measured using 40 different electrode pairs. The red line shows a normal distribution with mean 40 and standard deviation 20. (b) Change in resistance due to application of current pulses fitted to a sigmoid function, (c) Simulated resistance values of the NW network paths modeled using 10 discrete resistance states distributed along a sigmoid function.*

states were assumed to be nonlinearly distributed following a sigmoid function, consistent with the experimental observation of MR variation with current magnitude (Fig. S9 b, c).

For backpropagation, mean squared error was used as the cost function, defined as

$$C = \frac{1}{2}\sum\left(y_i^L - d_i^L\right)^2 \quad (1)$$

where y_i^L and d_i^L are the predicted and desired outcomes at the ith neuron in the output layer. The gradient of the cost function, or error, is expressed as

$$\delta_i^L = y_i^L - d_i^L \quad (2)$$

This error was then propagated backward to the previous layer, where the error at layer l is given by

$$\delta_i^l = W_{ij}\delta_j^{l+1}f'_{l+1}. \quad (3)$$

Here W_{ij} is the weight connecting the input node to the output node and f'_{l+1} is the gradient of the activation function. The weight update was then calculated as:

$$\Delta W_{ij} = \eta x_i^l \delta_j^{l+1} f'_{l+1} \quad (4)$$

where η is the training rate of the network and x_i^l is the input values of the layer. To improve convergence speed, the learning rate η was reduced by 10% after each epoch.

References:

1. Nickel and its alloys. *NBS Circular* **1924,** *100*.
